# Shortfalls in the global protected area network at representing marine biodiversity

**DOI:** 10.1038/srep17539

**Published:** 2015-12-03

**Authors:** Carissa J. Klein, Christopher J. Brown, Benjamin S. Halpern, Daniel B. Segan, Jennifer McGowan, Maria Beger, James E.M. Watson

**Affiliations:** 1 Centre for Biodiversity and Conservation Science, School of Geography Planning and Environmental Management, University of Queensland, St. Lucia, Queensland, 4072 Australia; 2 The Global Change Institute, University of Queensland, St. Lucia, Queensland, 4072 Australia; 3 Australian Rivers Institute, Griffith University, Nathan, Queensland 4111 Australia; 4 Imperial College London, Silwood Park Campus, Buckhurst Road, Ascot SL57PY, UK; 5 The Bren School of Environmental Science and Management, University of California, Santa Barbara, CA 93117, USA; 6 National Center for Ecological Analysis & Synthesis, Santa Barbara, CA 93101, USA; 7 Wildlife Conservation Society, Global Conservation Program, Bronx, NY 10460, USA; 8 Centre for Biodiversity and Conservation Science, School of Biological Sciences, University of Queensland, St. Lucia, Queensland, 4072 Australia

## Abstract

The first international goal for establishing marine protected areas (MPAs) to conserve the ocean’s biodiversity was set in 2002. Since 2006, the Convention on Biological Diversity (CBD) has driven MPA establishment, with 193 parties committed to protecting >10% of marine environments globally by 2020, especially ‘areas of particular importance for biodiversity’ (Aichi target 11). This has resulted in nearly 10 million km^2^ of new MPAs, a growth of ~360% in a decade. Unlike on land, it is not known how well protected areas capture marine biodiversity, leaving a significant gap in our understanding of existing MPAs and future protection requirements. We assess the overlap of global MPAs with the ranges of 17,348 marine species (fishes, mammals, invertebrates), and find that 97.4% of species have <10% of their ranges represented in stricter conservation classes. Almost all (99.8%) of the very poorly represented species (<2% coverage) are found within exclusive economic zones, suggesting an important role for particular nations to better protect biodiversity. Our results offer strategic guidance on where MPAs should be placed to support the CBD’s overall goal to avert biodiversity loss. Achieving this goal is imperative for nature and humanity, as people depend on biodiversity for important and valuable services.

The health of the ocean is jeopardized by human activities^1^,^2^ and it is widely accepted that marine protected areas (MPAs) are a fundamental strategy for protecting marine biodiversity^3^,^4^. Over the past decade, the Convention on Biological Diversity (CBD) has driven the marine conservation agenda^5^, resulting in the greatest surge in MPA establishment in history^6^. A critical part of assessing the achievement of CBD targets and informing future placement of protected areas are ‘gap analyses’, or spatial analyses that determine the extent to which species and ecosystems are represented in existing protected areas^7^,^8^. Such information is grossly incomplete for the ocean, resulting in an uninformed debate about global priorities for MPA establishment.

While some marine gap analyses suggest that existing MPAs are inadequate for protecting biodiversity^9^, they are almost always limited to single nations. Global marine gap analyses found similar results, but only focused on large ecoregions^10^, particular ecosystems^10^,^11^, or limited numbers of species^12^, giving little insight into how well marine species diversity is represented in the global MPA network.

Using 2014 data from the World Database on Protected Areas (WDPA)^13^ and a novel dataset of distribution maps for 17,348 marine fishes, mammals, and invertebrates^14^, we conducted the first comprehensive global gap analysis to determine the representation of marine species in MPAs. We considered a species to be at least partially ‘covered’ if a protected area overlapped with any part of its distribution^7^. If no overlap exists, then the species is a ‘gap species’. We conducted the analysis using four different subsets of protected areas to account for different interpretations for what constitutes an MPA: 1) spatial overlap with any marine area 2) identified as ‘marine’ in the WDPA database 3) identified as ‘marine’ in the WDPA database and classified as IUCN I-IV; 4) spatial overlap with any marine area and classified as IUCN I-IV (2,967,898 km^2^) (see Supplementary Information for more detail).

## Results and Discussion

**Figure 1:**
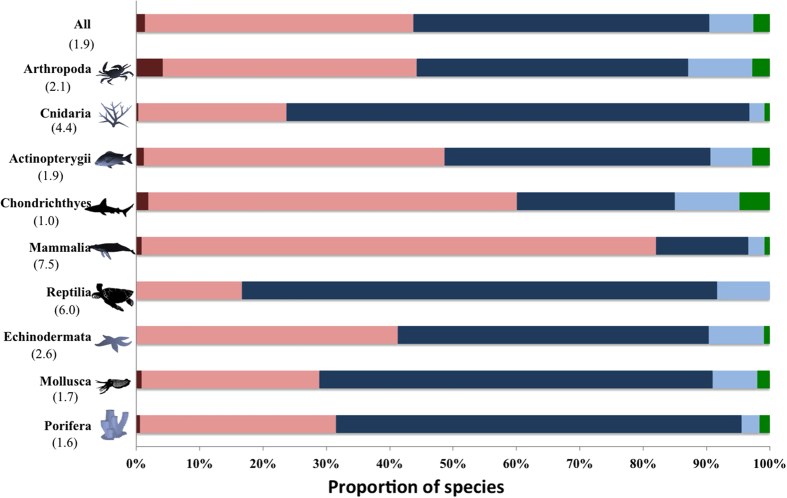
Percentage of marine species with 0% (dark red), 0–2% (pink), 2–5% (dark blue), 5–10% (light blue), and >10% (green) of their range overlapping with marine protected areas (IUCN I-IV). Data are shown for all species (top) and species in the 6 largest phyla (n = 16181), where the largest phyla (Chordata) is split into its 4 largest classes (Actinopterygii, Chondrichthyes, Mammalia, Reptilia). Median range size (million km^2^) of species in each group is shown in brackets. Created by CJK using Microsoft Excel and Powerpoint Software. Tracey Saxby, Integration and Application Network, University of Maryland Center for Environmental Science (ian.umces.edu/imagelibrary/).

**Figure 2:**
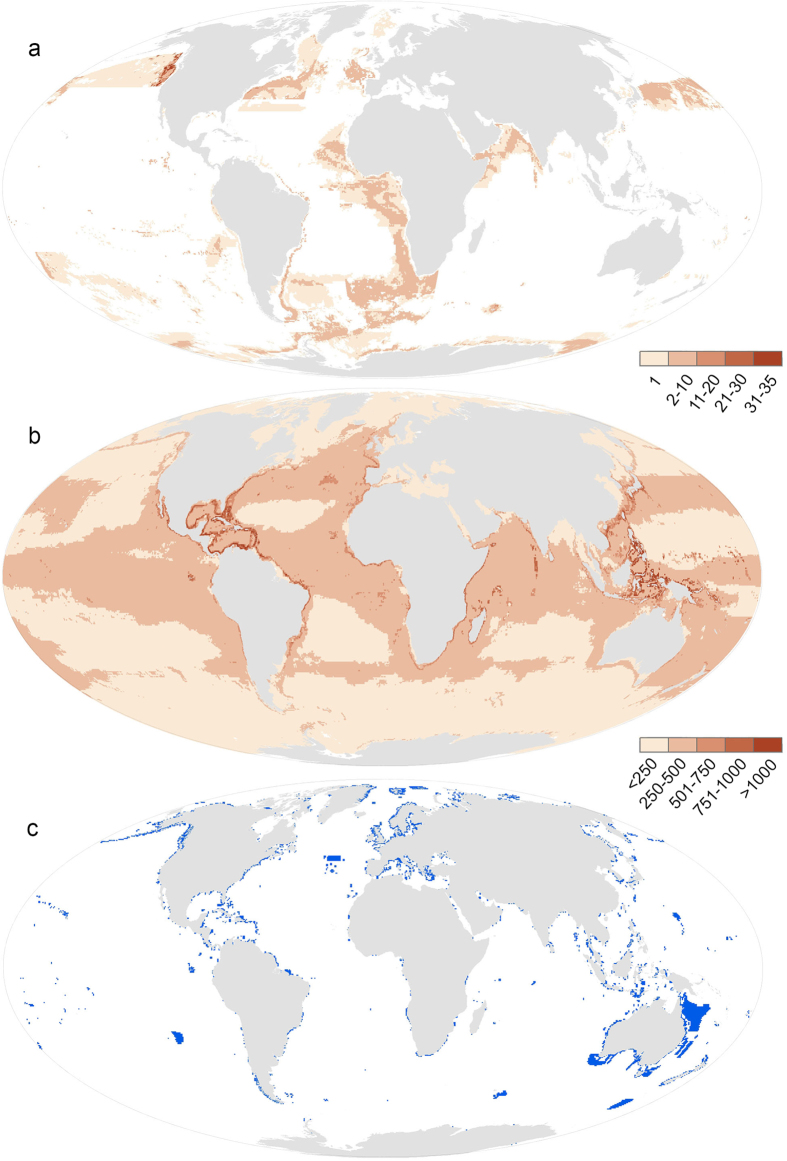
Density map of gap species (**a**) and very low coverage species (<2% of range represented) (**b**) per half-degree cell, created by overlaying the ranges of all species in these groups. Marine protected areas classified as IUCN I-IV that spatially overlapwith any marine area are shown in blue (**c**). Density maps using other marine protected area classifications are shown in Figures S1 and S2. Created by JM using ESRI ArcGIS Software.

We report results on all four subsets of protected area data (Table 1), but focus on the findings produced using subset 4: protected areas that spatially overlap with any marine area that are classified in stricter conservation classes (IUCN I-IV). Using this subset, we identified 245 (1.4%) gap species globally (Fig. 1, Table 1), 95% of which are found inside national exclusive economic zones and are primarily in temperate waters and away from coastlines (Fig. 2a, Fig. S1). Places with the greatest number of gap species are the USA, Canada, Brazil, and Antarctica (including Kerguelen Islands); strategically placing protected areas in these places could halve the number gap species. Interestingly, areas near the coastline do not contain gap species (Fig. 2). In some places this is due to the existence of many coastal MPAs, such as in Australia. However, in places where there are fewer MPAs, such as Argentina, this may be because there are species with large ranges that overlap with a protected area elsewhere (Fig. 2). The threat status of 17 of the 245 gap species have been assessed by the IUCN; five are threatened, including the critically endangered *Phocoena sinus* (Vaquita, a porpoise endemic to the Gulf of California that is heavily impacted by bycatch from fishing).

**Figure 3:**
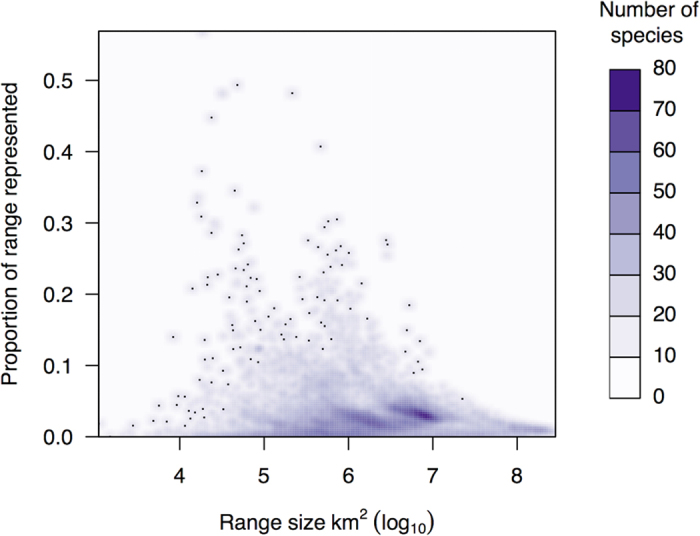
Overlap of marine species with protected areas (IUCN I-IV) by species range size (≥0.5 probability of species occurrence).

An analysis of gap species only provides one element of how well MPAs protect biodiversity. When we considered the degree of species coverage, we uncovered dramatic shortfalls that suggest current MPAs (IUCN I-IV, overlapping with any marine area) are not effective at conserving marine biodiversity. Overall, 7,584 (43.7%) species had very low (<2% of range represented), 15,693 (90.5%) had low coverage (<5% of range represented), and 16,897 (97.4%) had less than 10% of their range represented (Table 1). There was clear variation in the degree of coverage across taxonomic groups (Fig. 1) and species’ range size (Fig. 3). Given that less than 1% of the ocean contains an IUCN category I-IV MPA, species with large ranges (e.g., Mammalia) tend to have very low coverage (Fig. 3). Species with very low coverage are concentrated in tropical coastal areas (Fig. 2), suggesting that these MPAs are not designed to adequately represent their biodiversity. Given a goal of the CBD is to increase coverage of species in MPAs globally, our results indicate that conservation investment should be directed to countries within regions that have the lowest coverage (e.g., The Coral Triangle, Caribbean, North America, Japan) so that they can both increase and strategically place future MPAs. As nations have the authority to implement MPAs within their exclusive economic zones, serious commitments to enhancing marine protection can dramatically increase the coverage of species within MPAs.

Little information exists to guide decisions about adequate amounts of species ranges that need to be protected to ensure species persistence. However, marine conservation plans rarely, if ever, target less than 10% of any element of biodiversity (including species and habitats)^15^,^16^,^17^ and closing 10% of a species range to fishing has been recommended as the minimum necessary to obtain fisheries benefits^18^. Thus, the result that just 2.6% of species have 10% of their range covered with a protected area suggests a profound conservation shortfall (Fig. 1).

Even these sobering results likely overestimate how well MPAs represent species, because of several assumptions required when using deficient global data. First, we assumed that MPAs effectively protect biodiversity. However, the effectiveness of individual protected areas varies^11^ but is unknown at the global scale. Our analysis that considers all MPAs in the WDPA database is likely to contain a substantial amount of area that offer no or little protection to marine biodiversity. Ideally, we would exclude such areas in the gap analysis, but reliable information about the level of protection each offers is not consistently available. However, the WDPA data includes a field that indicates its IUCN protected area management category^19^, which is a classification for protected areas according to their management objectives, and is considered the global standard for defining and recording protected areas by the United Nations and many national governments.

The IUCN recognizes six categories of protected areas, ranging from category Ia (Strict Nature Reserve) with the strictest terms of management for preservation of biodiversity, to category VI (Protected Area with Sustainable Use of Natural Resources). When used in conservation planning, categories I-IV are generally considered to be the stricter conservation classes^7^ as classes V and VI permit most human activities (including mining and fishing)^20^, but even this is debatable and depends on what aspect of biodiversity is being proteced^21^,^22^. Thus, we we conducted the gap analysis with MPAs in IUCN categories I-IV for two of four scenarios (Table 1), assuming that human activities allowed in classes V and VI negatively impact the conservation of most species. However, we acknowledge that the IUCN classification is incomplete (e.g., about 3.6 million km2 of area has not been assigned a class) and inconsistent (pers communication B MacSharry) in the WDPA database, and that MPAs, regardless of classification, vary in the degree of effectiveness and enforcement^23^,^11^. In comparing our results using different subsets of MPAs, with and without IUCN classes considered, we found that the proportion of gap species only varied from 0.7–1.8%, and as expected, representation improved with total MPA area (Table 1).

Our results are subject to the limitations of global data available on marine species. We assumed that protection of all areas within a species’ range equally contribute towards its protection, and we do not take into account species specific traits, including ecological processes and areas important for different life-history stages (e.g., spawning aggregation sites, breeding grounds, feeding areas)^24^,^25^,^26^ that may require protection to ensure persistence. An element of uncertainty stems from the mismatched resolution of the protected area data (fine, <1 km^2^) and the species data (course, 0.5 degree grid). We assumed that the amount of a species’ range represented in an MPA was equal to the area of MPA within the grid; in cases where the actual species’ range is only a portion of the grid and does not overlap with MPAs, this will overestimate representation.

A further caveat to our results is the use of models to predict ranges in the absence of globally comprehensive observations of ranges. We chose to use predictions, because observations at a global scale are biased to regions where survey effort has been high^27^. This would likely underestimate representation of species’ ranges in poorly sampled regions. We test the sensitivity of our results to model predictions by varying the probability required for a species to be present from 0.00–1.0. We found that larger range sizes (i.e., lower probability thresholds) resulted in a lower proportion of gap species and less species with more than 10% of their range protected (Table 2). Comparing the threshold value of 0.5 with the least conservative estimate (i.e., 0), the difference in proportion of gap species and species with >10% of their range represented was 0.5% (87 species) and 1% (173 species), respectively. Thus, the global representation of species with the current distribution of protected areas is relatively insensitive to model uncertainties.

MPAs are not a panacea for conserving marine biodiversity^4^, and they can only contribute to biodiversity conservation if effectively managed. For some species, the best conservation outcome may be achieved with other strategies, including fisheries regulations^28^ and land-use management. Our results provide the first global baseline required to measure conservation progress and plan for future protected areas. This baseline is timely as we are halfway through the period for achieving CBD goals, and the recent outcome statement from the 6^th^ World Parks Congress (which convened over 6,000 people from 170 countries involved in protected area science and governance) called for nations to act urgently to make progress on their commitments^29^. As the global MPA network grows, the baseline we have created should be updated regularly and be used to measure how well nations are achieving CBD goals, including establishing MPAs in ‘areas of particular importance for biodiversity’ (Aichi target 11) and improving the status of biodiversity by safeguarding species (Strategic Goal C)^30^. While our coverage species richness maps (Fig. 3; Figs S1 and S2) can help strategically guide broad conservation investment, they alone are not sufficient to guide placement of new protected areas^31^. It is imperative that new MPAs are systematically identified and take into account socioeconomic costs of implementation, feasibility of success, other aspects driving biodiversity (e.g., bioregions), and complementarity metrics^32^,^33^. As most biodiversity remains poorly represented, the task of implementing an effective network of MPAs, in addition to other conservation measures, is urgent.

## Methods

### Data

Data on the global distribution of protected areas were obtained from the 2014 World Database on Protected Areas^13^. This database is the most comprehensive database on protected areas, yet it still contains inaccuracies and/or inconsistencies (see Supplementary Information)^34^. To account for some of these, we used four different subsets of the data, each of which produced a different version of the global marine protected area estate (Table 1 and Supplementary Information). We used modeled species distribution data for 17,348 marine species derived from AquaMaps^14^, a species distribution modeling tool that produces standardized global range maps of aquatic species. This is the most comprehensive and highest resolution data available on the distribution of marine biodiversity globally, and includes Animalia (fishes, marine mammals, and invertebrates), Plantae (fleshy algae, seagrass), Chromista (calcifying algae) and Protozoa. The species distribution maps predict relative probabilities of species occurrence (ranging from 0.00–1.00) at a resolution of 0.5 degree cells. It is assumed that the preferred range is where probability is 1, outside the range limits is where probability is 0, and between these two thresholds the relative environmental suitability decreases linearly^11^.The focus of our results are using a probability threshold value of 0.5 or greater. As there is no recommended threshold to use^12^, we test the sensitivity of our results to uncertainty in the model predictions by varying the threshold for the probability of occurrence (Table 2).

### Gap analysis

The MPA data (fine, <1 km^2^) were aggregated to 0.5 degree grids, to match the course resolution of the species range maps. We determined the proportion of protected area in each 0.5 degree grid. As we do not know the exact distribution of species within each grid, we assumed that the area of a species’ range represented in MPAs was equal to the area of MPA coverage for grid cells that species was present in. As such, we likely over-estimate representation of species in protected areas.

## Additional Information

**How to cite this article**: Klein, C. J. *et al.* Shortfalls in the global protected area network at representing marine biodiversity. *Sci. Rep.* **5**, 17539; doi: 10.1038/srep17539 (2015).

## Supplementary Material

Supplementary Information

## Figures and Tables

**Table 1:** Proportion of marine species with 0% (gap), 0–2%, 2–5%, 5–10%, and >10% of their range overlapping with protected areas, using four different subsets of protected areas from the World Database on Protected Areas^13^ (See Supplementary Information): 1) all; 2) identified as ‘marine’; 3) identified as ‘marine’ and classified as IUCN I-IV; 4) spatially cover a marine area and classified as IUCN I-IV. Species probability threshold was ≥0.5.

Protected areas	Area (km^2^)	Gap	Covered 0–2%	Covered 2–5%	Covered 5–10%	Covered >10%
1) All	10,064,935	0.7%	10.7%	17.5%	20.6%	50.6%
2) Marine	8,042,751	1.1%	12.9%	18.6%	22.8%	44.7%
3) IUCN I-IV, ‘Marine’	2,913,402	1.8%	43.9%	45.1%	6.6%	2.5%
4) IUCN I-IV, Spatially Marine	2,967,898	1.4%	42.3%	46.8%	6.9%	2.6%

**Table 2:** Proportion of marine species with 0% (gap), 0–2%, 2–5%, 5–10%, and >10% of their range overlapping with protected areas (IUCN I-IV) for species probability thresholds ranging from 0–1.

Species threshold	Median range size (km^2^)	Gap	Covered 0–2%	Covered 2–5%	Covered 5–10%	Covered >10%
0	2,953,998	0.9%	42.1%	49.0%	6.4%	1.6%
.2	2,453,986	1.1%	42.0%	48.1%	6.6%	2.1%
.4	2,080,061	1.3%	42.2%	47.2%	6.8%	2.4%
.5	1,900,421	1.4%	42.3%	46.7%	6.9%	2.6%
.6	1,718,444	1.6%	42.2%	46.4%	7.0%	2.8%
.8	1,333,229	2.0%	42.4%	44.9%	7.5%	3.2%
1	798,184	3.2%	42.9%	39.0%	10.6%	3.9%
