# Supplementary material for: Shortfalls in the global protected area network at representing marine biodiversity
Source: Sci Rep. 2015 Dec 3;5:17539. doi: 10.1038/srep17539 (PMC4668359; doi:10.1038/srep17539)
Supplement: Supplementary Information [file srep17539-s1.pdf]

Supplementary Information

Shortfalls in the global protected area network at representing marine biodiversity

Carissa J Klein[1*], Christopher J Brown[2,3], Benjamin S Halpern[4,5,6*], Daniel B Segan[7], Jennifer McGowan[8], Maria Beger[8], James EM Watson[1,7]

Supplementary Methods

The results shown in the main text of this manuscript were derived from a subset of data the World Database on Protected Areas[1] and AquaMaps[2]. Here, we describe the data processing decisions that we made to produce the results reported in the main text.

World Database on Protected Areas

This database is a compilation of protected areas from individual countries and, as a result, can explain some of the problems that we describe. Our results are subject to two main data processing decisions to do with the World Database on Protected Areas[1], both which impact the size and exact location of protected areas in the global MPA estate (Table 1).

<u>Marine versus terrestrial protected areas</u>

The database includes both terrestrial and marine protected areas, and includes a data field to identify protected areas that provide protection for the marine environment[3]. The data for this field are incomplete and there are MPAs that have not been classified as 'marine' that spatially overlap with the ocean. For example, the Mayotte MPA covers over 68,000 km^2 of the Indian ocean but is not listed as 'marine' in the database[4]. Thus, results that restrict the analysis to 'marine' using this data field underestimate the area of global MPAs (Table 1). However, the results of the gap analysis are similar regardless of whether a 'marine' filter was applied; the proportion of gap species is slightly higher and the proportion of species with greater than 10% of their range represented is slightly lower when a 'marine' filter is applied (Table 1).

With the aim of obtaining a more accurate representation of which protected areas are marine, we excluded the terrestrial portion of all protected areas that overlap with land for all gap analyses conducted[5]. However, the resulting data could result in errors of omission and commission. For example, errors of commission could be introduced when the spatial extent of a protected area that is entirely terrestrial, extends beyond the coastline, causing us to overestimate marine protection. Whereas errors of omission could be introduced if the spatial extent of a MPA falls entirely on the land, which is likely the case for some small coastal MPAs, causing it to be excluded from the MPA estate.

IUCN Classifications

The World Conservation Union (IUCN) has a classification for protected areas according to their management objectives, and is considered the global standard for defining and recording protected areas by the United Nations and many national governments. The IUCN recognizes six categories of protected areas, ranging from category Ia (Strict Nature Reserve) with the strictest terms of management for preservation of biodiversity, to category VI (Protected Area with Sustainable Use of Natural Resources). The database includes a field that indicates its IUCN protected area management category and its recommended use in conservation planning[3]. When used in conservation planning, categories I-IV are generally considered to be the stricter conservation classes[6] as classes V and VI permit most human activities (including habitation, mining and all types of fishing)[7], but even this is debatable and depends on what aspect of biodiversity is being protected[4,8]. However, three main factors that complicate category level analysis of the MPA estate are (pers communication B MacSharry): 1) the current guidelines for the categories were

released in 2008 and not all countries have re-evaluated pre-2008 protected areas; 2) interpretation of the guidelines is inconsistent between countries[9]; 3) not all protected areas have been assigned a IUCN class, including to 3,619,857 km2 of possible MPAs. Given this, we conduct the gap analysis with all protected areas, regardless of IUCN classification (Table 1). As the MPA estate is much larger when all protected areas are included, it is no surprise that representation improves as the size of the MPA estate used for the assessment increases (Table 1). A density map of gap species and species with <2% of their range protected, for each protected area definition, is provided in Figures S1 and S2.

References

1. IUCN and UNEP-WCMC. *The World Database on Protected Areas (WDPA): [On-line], April 2014*. Cambridge, UK: (UNEP-WCMC, 2014). Available at www.protectedplanet.net.

2. Kaschner, K. *et al.* AquaMaps: Predicted range maps for aquatic species. *Version 2014* (2014). at <www.aquamaps.org>

3. UNEP-WCMC. Data Standards for the World Database on Protected Areas. (2014).

4. Dudley, N., Parrish, J. D., Redford, K. H. & Stolton, S. The revised IUCN protected area management categories: the debate and ways forward. *Oryx* **44,** 485–490 (2010).

5. Wessel, P. & Smith, W. H. F. GSHHG - A Global Self-consistent, Hierarchical, High-resolution Geography Database (Version 2.2.4). (2013).

6. Rodrigues, A. S. L. *et al.* Effectiveness of the global protected area network in representing species diversity. *Nature* **428,** 640–643 (2004).

7. Day, J. *et al. Guidelines for applying the IUCN Protected Area Managment Categories to Marine Protected Areas*. 36 (2012).

8. Boitani, L. *et al.* Change the IUCN protected area categories to reflect biodiversity outcomes. *PLoS Biol.* **6,** e66 (2008).

9. Leroux, S. J. *et al.* Global protected areas and IUCN designations: Do the categories match the conditions? *Biol. Conserv.* **143,** 609–616 (2010).

Supplementary Figures

Figure S1. Density map of gap species per half-degree cell, created by overlaying the ranges of species (threshold ≥.5) with all protected areas (a), protected areas classified as 'marine' (b), protected areas classified as IUCN I-IV and marine (c). Created by JM using ESRI ArcGIS Software.

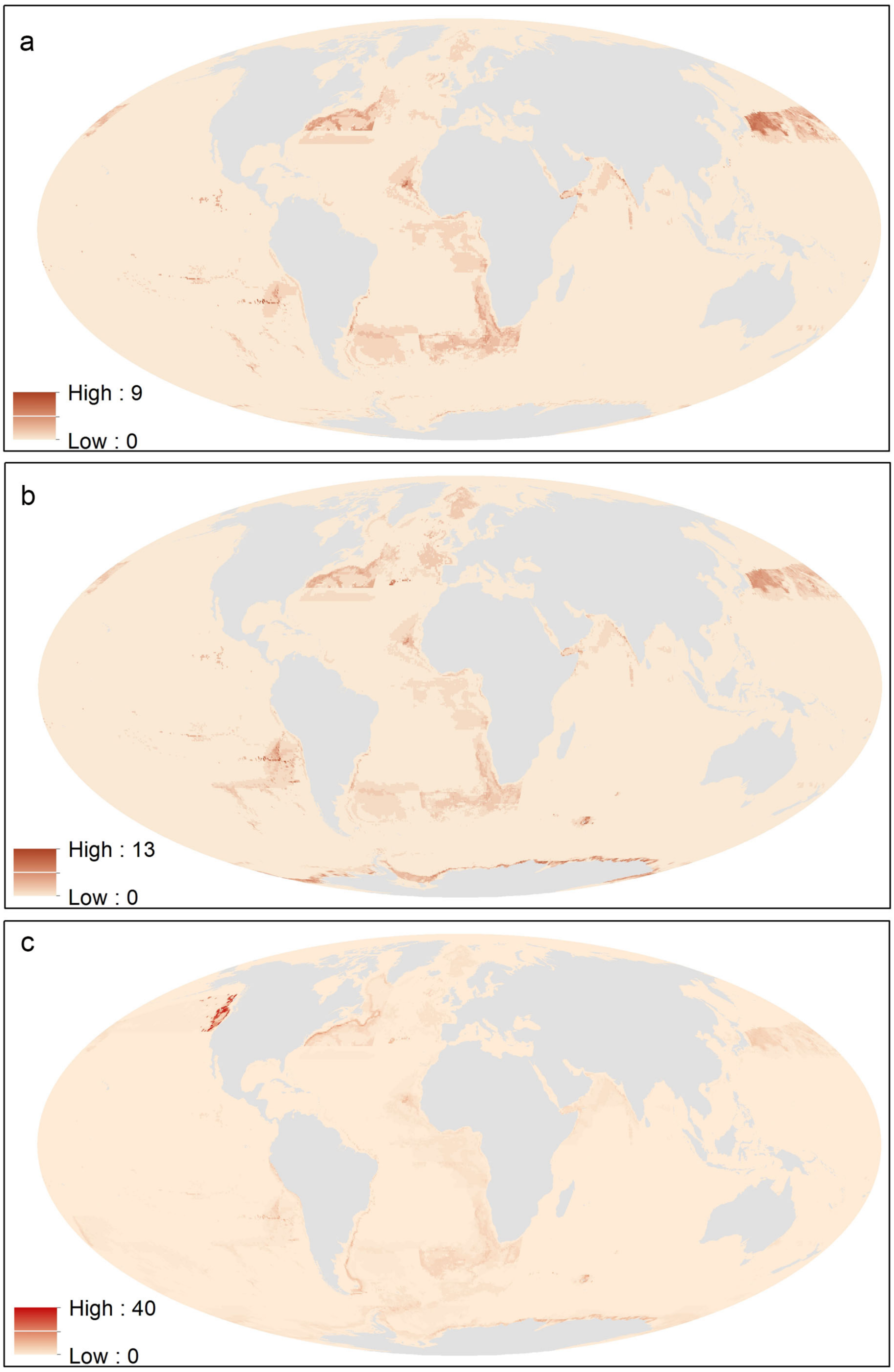

Figure S2. Density map of species with very low coverage (<2% of range represented) per half-degree cell, created by overlaying the ranges of species (threshold ≥.5) with all protected areas (a), protected areas classified as 'marine' (b), protected areas classified as IUCN I-IV and marine (c). Created by JM using ESRI ArcGIS Software.

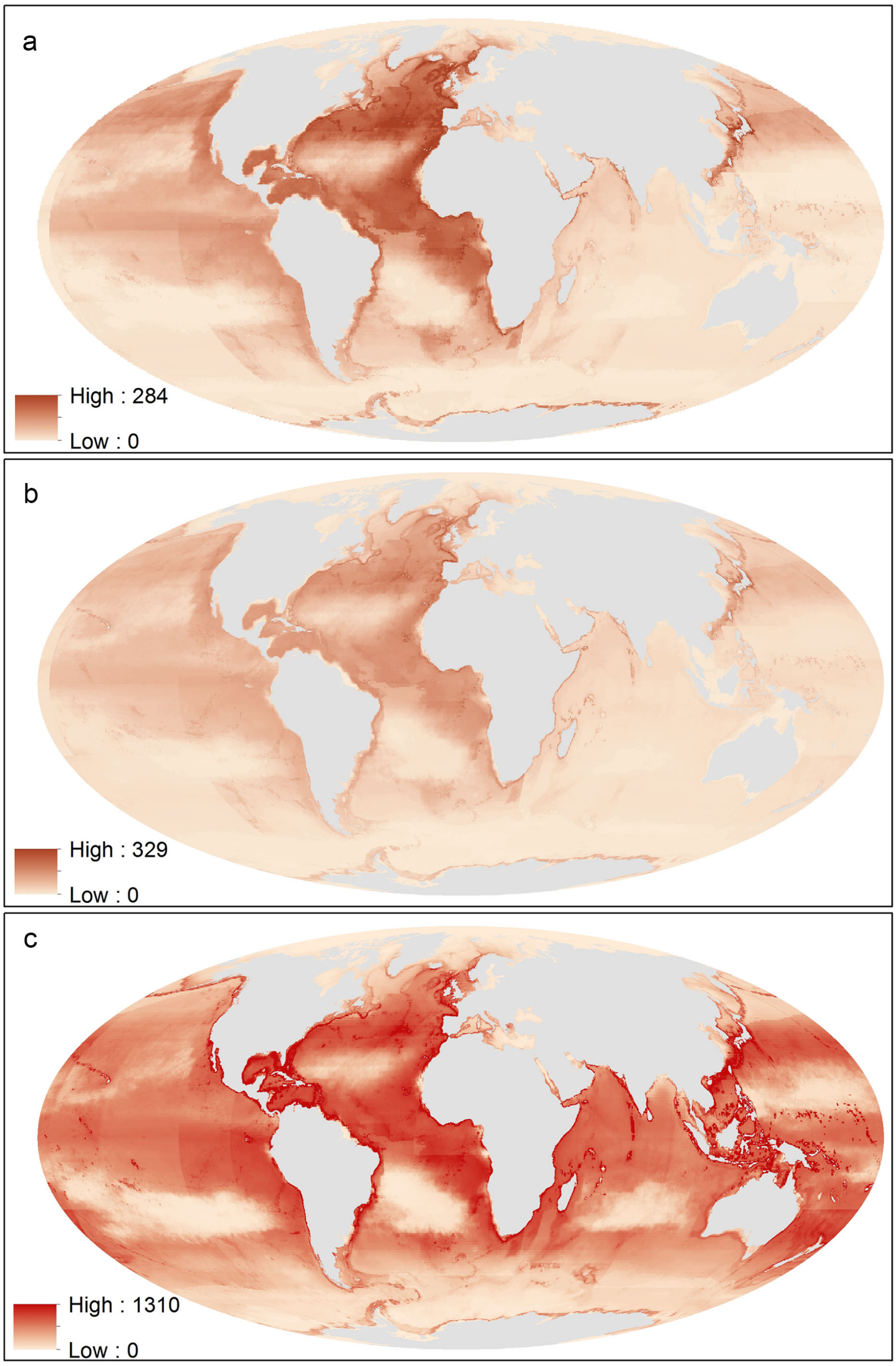
a
High : 284
Low : 0
b
High : 329
Low : 0
c
High : 1310
Low : 0
